# A DTI study of leukoaraiosis and the differential diagnosis between leukoaraiosis and acute lacunar infarction

**DOI:** 10.1111/cns.13191

**Published:** 2019-07-11

**Authors:** De‐Qiang Zhao, Zhan‐Wen Wang, Ying Cheng, Zhen Yuan, Frédérique Rene, Haoyi Liu, Artem Pliss, Ping Luan

**Affiliations:** ^1^ Department of Neurology Nanfang Hospital of Southern Medical University Guangzhou China; ^2^ Medical Center Shenzhen University Health Science Center Shenzhen China; ^3^ Faculty of Health Sciences University of Macau Macau China; ^4^ INSERM, U1118, Mécanismes Centraux et Péripheriques de la Neurodégénérescence Strasbourg France; ^5^ Institute for Laser, Photonics and Biophotonics State University of New York Buffalo NY USA

Leukoaraiosis describes diffuse white matter abnormalities on CT or MR brain scans, often seen in the normal elderly and in association with vascular risk factors such as hypertension, or in the context of cognitive impairment. Leukoaraiosis (LA) was first and foremost introduced as an imaging diagnostic terminology in 1987. LA describes the neuroimaging abnormalities of the white matter around lateral ventricle and centrum semiovale that appears as diffuse, punctate, continuous, or patchy. The study found that computed tomography (CT) and routine magnetic resonance imaging (MRI) can see LA lesions number, location, and degree of damage. However, the degree of lesions is seldom consistent with clinical manifestations. Many patients with LA do not present with any clinical symptoms. Nonspecific changes around ventricle on CT or MRI do not have necessarily clinical significance.^1^ Diffusion tensor MR imaging (DTI) is currently the only noninvasive method in living human brain tissue, which can display the information of the course,^2^ direction, arrangement, tightness, and myelination of the white matter fiber tracts.^3^, ^4^ The DTI can be used to explore the parameters variation in the patients with LA and explore differential diagnostic value between LA and acute Lacunar infarction (LI).

The objects of study were selected from the Nanfang hospital and the first affiliated hospital of Shenzhen university. LA patients were classified according to Wahlund LA classification standard. All patients were subjected to MRI and DTI scan. DTI data import GE workstation (AW4.3) and DTI postprocessing program with Functool software for processing. All data were statistically processed using SPSS 11.5 software. Fractional anisotropy (FA) values and mean diffusivity (MD) data were expressed as Mean ± SD. The significance of the difference between two groups was determined with two‐tailed Student's *t* test. Multiple comparisons were analyzed by analysis of variance (ANOVA) followed by Fisher's least significant difference (Fisher's LSD) *post hoc* test. Differences with *P‐*values < 0.05 were considered statistically significant.

The results showed that in the different anatomical parts of the hemispheres, MD values in lesions of patients with LA more increased than normal appearing white matter (NAWM) area and the control group, respectively, FA values more reduced than NAWM area and the control group, respectively. Compared with control, MD values in NAWM area of patients with LA were increased while FA values were decreased (Table 1). At the same time, the degree of LA is heavier, the higher the value of MD, were positively correlated; LA degree heavier, the FA values lower and a negative correlation (Table 1, the LA grade shows in Figure 1A‐C). The changes in DTI parameters of cerebral white matter in the patients with LA are closely related to the pathogenesis and pathology basis of LA.^5^

**Table 1:** Mean diffusivity (MD) and fractional anisotropy (FA) of white matters in different severe grades of Leukoaraiosis (LA) sufferers and control group

group	Around the front lateral ventricle		Around lateral ventricle back end		Centrum semiovale	
	MD	FA	MD	FA	MD	FA
Control group (n = 120)	0.76 ± 0.06	0.41 ± 0.03	0.73 ± 0.04	0.44 ± 0.04	0.71 ± 0.03	0.40 ± 0.03
LA‐1 (n = 45)	0.96 ± 0.05	0.35 ± 0.04	0.95 ± 0.06	0.37 ± 0.03	0.93 ± 0.04	0.34 ± 0.05
LA‐2 (n = 45)	1.04 ± 0.08	0.33 ± 0.05	1.02 ± 0.07	0.35 ± 0.06	0.97 ± 0.05	0.32 ± 0.04
LA‐3 (n = 30)	1.18 ± 0.05	0.27 ± 0.03	1.15 ± 0.07	0.29 ± 0.04	1.12 ± 0.05	0.28 ± 0.03
*F*	106.419	24.901	109.503	27.039	184.321	15.848
*P*	0.000	0.000	0.000	0.000	0.000	0.000

**Figure 1:**
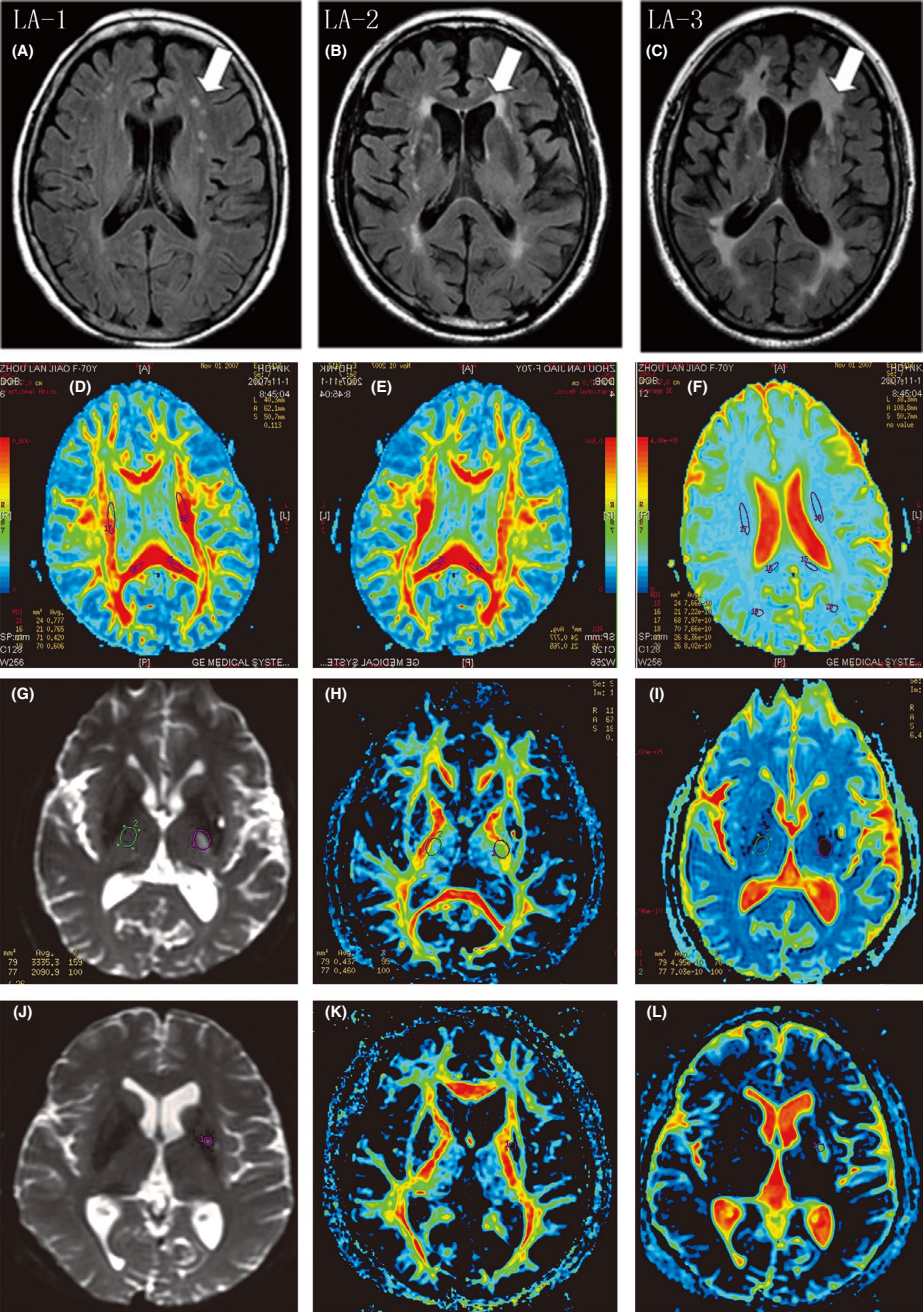
A, LA‐1 Focal lesions, around before, during, and back of the cerebral ventricles white matter see scattered punctate limitations lesions. B, LA‐2 Ventricles lesions began to merge connection, around before, during, and back of the cerebral ventricles white matter see the fusion or partial fusion patchy lesions. C, LA‐3 Pathological changes into flake, diffuse involvement of white matter around the lateral ventricle, with or without U fiber involvement. D‐F, FA and MD figure in patient with LA. Panel D and Panel E, basal ganglia level FA figure (red area has high anisotropy). Panel F, basal ganglia level MD figure (red area with a high degree of diffusion). G‐I, patient with lacunar cerebral infarction, male, 66 years old. Panel G, basal ganglia level T2WI. Panel H, basal ganglia level FA figure. Panel I, basal ganglia level MD figure. J‐L, patient with lacunar cerebral infarction, male, 52 years old. Panel J, basal ganglia level T2WI. Panel K, basal ganglia level FA figure. Panel L, basal ganglia level MD figure

This research shows on MRI that the normal white matter area had MD value higher than the control group, while FA values were lower. These results are consistent with those obtained by Sullivan, etc In addition, our study also found that around front‐end and back‐end lateral ventricle of patients with LA, the MD value in NAWM of their left and right hemisphere have significant difference. In addition, NAWM in LA area also has progressive cerebral white matter lesions and may develop into LA. Only the irregular changes of these nerve fibers and the slight pathological changes cannot be detected by conventional MRI. The results confirmed that the DTI is more sensitive than conventional MRI to monitor cerebral white matter lesions. Therefore, DTI can detect early LA lesions and show the value of DTI in LA diagnosis, research, development and change in LA and judgment effect of LA treatment.

Lacunar infarction and LA have similar pathological basis, and they have very similar imaging findings on conventional MRI, so differential diagnosis between them is difficult.^6^ Lacunar cerebral infarction is due to small artery blockages ischemia and necrosis. 7 DTI can detect acute ischemic focal within 6 hours after ischemic cerebral stroke, even found MD lower at 105 minutes after ischemia attack.^8^ The data show infarction center of patients with LI (MD: 0.40 ± 0.05, FA: 0.35 ± 0.03), respectively, compared with the healthy side and the control group (MD: 0.73 ± 0.03, FA: 0.42 ± 0.02), and both MD and FA values were decreased. On the corresponding contralateral position of LI (MD: 0.70 ± 0.05, FA: 0.40 ± 0.05), MD and FA values were lower compared with control group, and MD (1.08 ± 0.03) values were increased in LA lesion area compared with controls while FA (0.32 ± 0.02) values were decreased (Figure1D‐L). These differences were statistically significant.

DTI studies have found that for different stages of LI, corresponding MD and FA values present the specificity of the three stages of change: MD decrease, FA increase; MD and FA both decrease; MD increase, FA decrease. Acute LI infarcts group MD and FA values than the control group and the healthy side were both decreased, respectively, MD and FA values at LI acute stage (the phase 2) were lower. In the acute stage of LI, infarcts brain tissue in phase cytotoxic edema or early vascular source edema, MD values significantly reduced, with the development of cerebral infarction, vascular endothelial cell damage, cell permeability increase, intercellular water accumulated lead to vascular source brain edema, water molecules diffuse ability further fall, the MD value further reduce. At the same time, because of the cellular structure damage and the loss of the tissue microstructure organization, anisotropy decreased significantly and FA values decreased. MD values of LI infarcts were significantly below these of LA lesions due to the decrease in total free‐water molecules diffusion in ischemia area. In addition, FA values of LI infarcts were obviously higher than that of LA lesions, often prompts the anisotropy of the former more than the latter. The characteristics of magnetic resonance DTI imaging of LI and LA is possible used for the differential diagnosis of the two.

Altogether, we showed that DTI is more sensitive than the MRI and can detect the early lesions which appear normal in MRI. Moreover, there is a characteristic difference in the DTI parameters between LA and LI.

## CONFLICT OF INTEREST

The authors declare no conflict of interest.
